# Cetuximab-induced skin exanthema: prophylactic and reactive skin therapy are equally effective

**DOI:** 10.1007/s00432-013-1483-4

**Published:** 2013-08-07

**Authors:** Thomas C. Wehler, Claudine Graf, Markus Möhler, Jutta Herzog, Martin R. Berger, Ines Gockel, Hauke Lang, Matthias Theobald, Peter R. Galle, Carl C. Schimanski

**Affiliations:** 1 Third Department of Internal Medicine, Johannes Gutenberg University of Mainz, Mainz, Germany; 2 First Department of Internal Medicine, Johannes Gutenberg University of Mainz, Mainz, Germany; 3 Toxicology and Chemotherapy Unit, German Cancer Research Center (DKFZ), Im Neuenheimer Feld 581, 69120 Heidelberg, Germany; 4 Department of General and Abdominal Surgery, Johannes Gutenberg University of Mainz, Mainz, Germany; 5 Department of Internal Medicine, Marienhospital Darmstadt, Martinspfad 72, 64285 Darmstadt, Germany

**Keywords:** Skin, Rash, Exanthema, EGFR, Cetuximab, Therapy reactive

## Abstract

**Purpose:**

Treatment with cetuximab is accompanied by the development of an acneiform follicular skin exanthema in more than 80 % of patients. Severe exanthema (grade III/IV) develops in about 9–19 % of patients with the necessity of cetuximab dose reduction or cessation.

**Methods:**

The study presented was a retrospective analysis of 50 gastrointestinal cancer patients treated with cetuximab in combination with either FOLFIRI or FOLFOX. One cohort of 15 patients received an in-house reactive skin protocol upon development of an exanthema. A second cohort of 15 patients received a skin prophylaxis starting with the first dose of cetuximab before clinical signs of toxicity. A third historic group of 20 patients had received no skin prophylaxis or reactive treatment.

**Results:**

19/20 patients of the historic group developed a skin exanthema. Grade III/IV exanthema was observed six times. Forty percent discontinued cetuximab therapy. The average time to exanthema onset was 14.7 days. Applying the reactive skin protocol after the first occurrence of an exanthema, the exanthema was downgraded as follows: No patients developed grade IV° exanthema, and two patients developed a grade II/III exanthema. In the majority of cases, the reactive skin protocol controlled the exanthema (grade 0–I°). No dose reductions in cetuximab were necessary. Applying the prophylactic skin protocol starting at the beginning of cetuximab application was not superior to the reactive skin protocol.

**Conclusions:**

Cetuximab-induced skin exanthema can be coped with a reactive protocol equally effective as compared to a prophylactic skin treatment. A prospective study with higher patient numbers is planned.

## Introduction

The inhibition of growth factor signaling pathways has proven to be an effective therapeutic option in a quite variety of tumor entities. Epidermal growth factor receptor (EGFR) inhibitors, for instance, are used in the treatment of colorectal cancer, head and neck cancer, non-small cell lung cancer, and pancreatic cancer among other malignancies (Ciuleanu et al. 2012; Saltz et al. 2004; Luedke et al. 2012; Busam et al. 2001) and are in general well tolerated. However, EGFR inhibitors, such as cetuximab, have the potential to induce a skin exanthema of follicular origin that occurs in the majority of patients (Ciuleanu et al. 2012). This exanthema has an acneiform appearance (Busam et al. 2001) and can present itself as maculae, papulae, or small pustulae occurring on the face, décolleté, or back. In addition, it may be accompanied by symptoms such as burning or itching.

Usually, it takes up to 2 or 3 weeks after the first intake of the EGFR inhibitor until the first signs of exanthema. Most patients only suffer from mild exanthemas (grade I° and II°). Nevertheless, about a fifth of the patients will experience severe exanthemas (grade III° or IV°). More severe or persistent exanthemas are eventually seen when EGFR antibodies are administered (Busam et al. 2001). By the occurrence of a severe exanthema, pause or dose reduction of the EGFR inhibitor can be considered. Discontinuation is necessary in approximately 10 % of patients (Busam et al. 2001). However, as patients are told that the development and intensity of an exanthema are rather associated with a better prognosis, the withdrawing of EGFR inhibitors from cancer therapy can be perturbing for the respective patients (Stintzing et al. 2012). Male gender and age below 70 years are considered as risk factors for the development of severe exanthemas (Lacouture et al. 2011).

Bothersome for the concerned patients, there is no standardized therapy for the therapy of skin toxicities. They are mostly coped with known strategies established for the therapy of acne. In the recent past, first randomized trials analyzed the prophylaxis and therapy of cetuximab-induced skin exanthemas (Lacouture et al. 2011; Scope et al. 2007; Jatoi et al. 2008).

Less severe and less symptomatic skin exanthemas were reported by a prophylactic intake of oral minocycline (Scope et al. 2007). However, this effect was only temporary and vanished in the long run. Similar results were reported by Jatoi et al. They analyzed the prophylactic effect of an oral tetracycline application on the development of skin exanthemas grade ≥II°. Efficacy was observed during the first weeks but not in a long-term setting (Scope et al. 2007). The mentioned antibiotics are commonly used for the treatment of acne. The clinical similarity between classical acne and an EGFR inhibitor-induced skin exanthema suggests that this medication might be successfully used for prevention or treatment of drug-induced exanthemas (Gammon et al. 1986; Meynadier and Alirezai 1998). However, the anti-inflammatory effects of tetra-/doxy-/and minocycline might also support exanthema palliation (Meynadier and Alirezai 1998; Fujita et al. 2007).

Another randomized phase II study analyzed the effect of a prophylactic versus reactive therapy during second-line panitumumab therapy in colorectal cancer (Lacouture et al. 2010). The prophylactic therapy including lotion, oral doxycycline, topical hydrocortisone (1 %), and sun block resulted in a significant reduction in skin exanthemas grade ≥II°. However, weak points in this study were the topical administration of topical cortisone in the prophylactic arm and the undefined reactive treatment regimen.

In view of these data, we retrospectively compared three patient populations who had received cetuximab therapy with either no standard skin treatment (historic group), or an in-house reactive skin protocol that was based on the therapeutic options of classical acne and treatment recommendations of the cetuximab manufacturer, or a prophylactic treatment with cleansing syndet, topical metronidazole ointment, and doxycycline 100 mg twice per day. We were able to perform this retrospective analysis, as we had established a reactive skin protocol and a prophylactic skin protocol several years ago and documented all skin toxicities according to National Cancer Institute’s Common Terminology Criteria for Adverse Events version 3.0 (NCI CTCAE v3.0) and digital photography. The decision whether a patient received reactive therapy or a prophylactic therapy was left to the patient.

## Methods

### Patients

Due to a lack of standardized guidelines, we stuck to an in-house reactive skin protocol and a prophylactic skin protocol derived from the common acne therapy, which was offered to all patients treated with cetuximab. The skin protocols were offered for the first time to a patient in April 2008. We retrospectively analyzed all patients receiving the reactive skin protocol under cetuximab-based chemotherapy starting from April 2008. However, we did not include the patient population reported earlier.

**Fig. 1:**
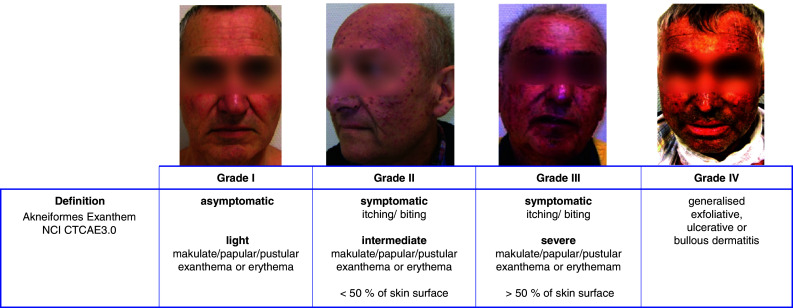
TKI-associated acneiform exanthema. TKI associated acneiform exanthema is classified according NCI CTCAE3.0

All adverse events were routinely documented on a weekly basis as per the NCI CTCAE v3.0 criteria. The evaluation entailed physical examination: a weekly assessment of patient performance status and weight; and an assessment of adverse events, including gastrointestinal toxicity and exanthema development. Skin toxicities were documented weekly by digital photography (Fig. 1).

### Reactive skin protocol

**Fig. 2:**
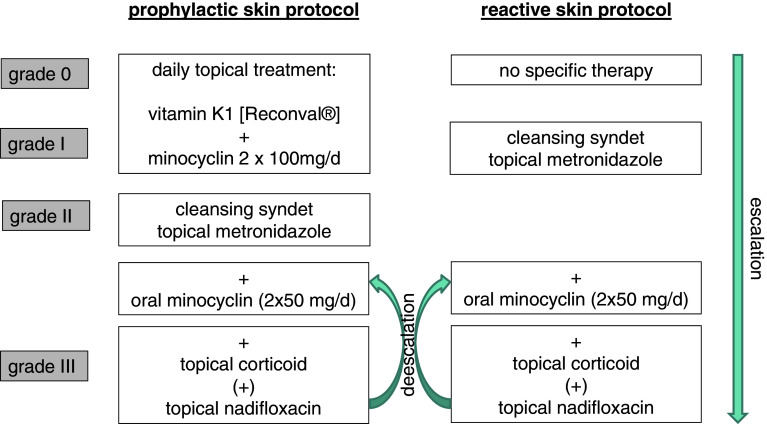
Prophylactic treatment regimen applied to group C and reactive treatment regimen applied to group B

The reactive skin protocol was established as follows: grade I° exanthema; topical cleansing syndet [Dermowas^®^]; and topical metronidazole cream (7.5 %) on affected skin areas [Rosiced^®^]. Grade II° exanthema: See grade I° treatment plus oral minocycline 50 mg twice daily. Grade III° exanthema: See grade II° treatment plus topical corticoid prednicarbat cream (0.25 %) on affected skin areas [Dermatop^®^] (Fig. 2). As soon as a grade III° had improved to grade ≤II°, application of topical corticoid was ceased.

It was specified that patients were to be withdrawn from minocycline in the event of ≥grade II°, nausea, and/or vomiting. The latter was characterized by two to five episodes of nausea/vomiting within 24 h.

### Prophylactic skin protocol

The prophylactic skin treatment protocol consisted of the application of a topical cleansing syndet [Dermowas^®^], a topical 7.5 % metronidazole ointment [Rosiced^®^], and doxycycline 100 mg (p.o.) twice per day (exanthema >II°; +topical corticoid prednicarbat cream (0.25 %) [Dermatop^®^]). In case of rash >grade II°, the skin treatment was identically performed as in the reactive skin protocol.

### Statistical analysis

Our data were explored in a retrospective descriptive setting using *t* test and *χ* ^2^-test.

## Results

### Patients

A total number of 50 patients were treated with cetuximab. Twenty patients of the historic cohort did not receive a standard skin treatment. Fifteen patients of the second cohort were treated according to our in-house reactive skin protocol starting in June 2008. Upon retrospective evaluation, all patients had received treatment under this protocol for a minimum of 12 weeks. In the third cohort, 15 patients received a prophylactic skin treatment consisting of a topical cleansing syndet [Dermowas^®^], a topical metronidazole ointment [Rosiced^®^], and doxycycline 100 mg (p.o.) twice per day. None of the patients had a history of acne. The retrospective analysis was conducted according to the requirements of the local ethics committee and was performed with the ethical standards laid down in the 1964 Declaration of Helsinki and its later amendments.

All patients suffered from a gastrointestinal adenocarcinoma stage UICC IV. All patients had a history of chemotherapy consisting of a standard initial cetuximab dose of 400 mg/qm and thereafter 250 mg/qm weekly combined with either irinotecan or platinum-based chemotherapy. None of the patient received radiation.

### Exanthema

**Fig. 3:**
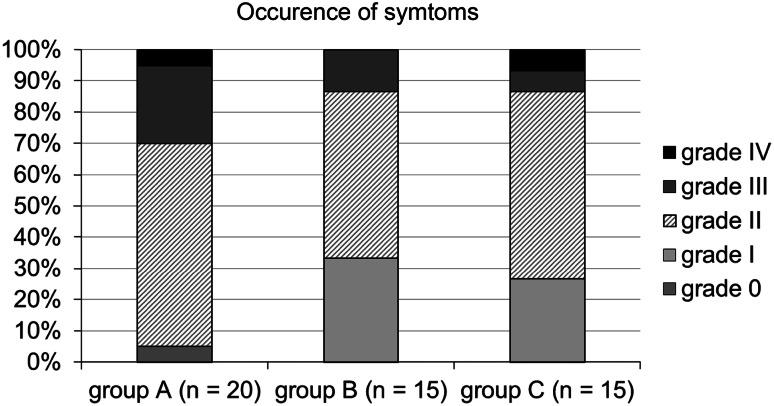
Occurrence of symptoms. Occurrence of maximum acneiform exanthema in the historic cohort A compared to the “reactive treatment” cohort B and “prophylactic treatment” group C

**Fig. 4:**
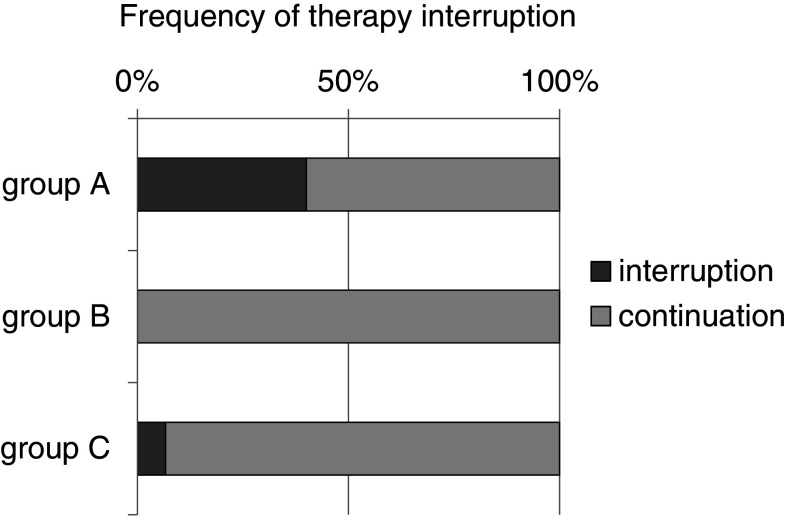
Frequency of therapy interruption. The “historic” cohort shows a frequency of 40 % therapy interruption compared to 0 % in cohort B and 7 % in cohort C

**Fig. 5:**
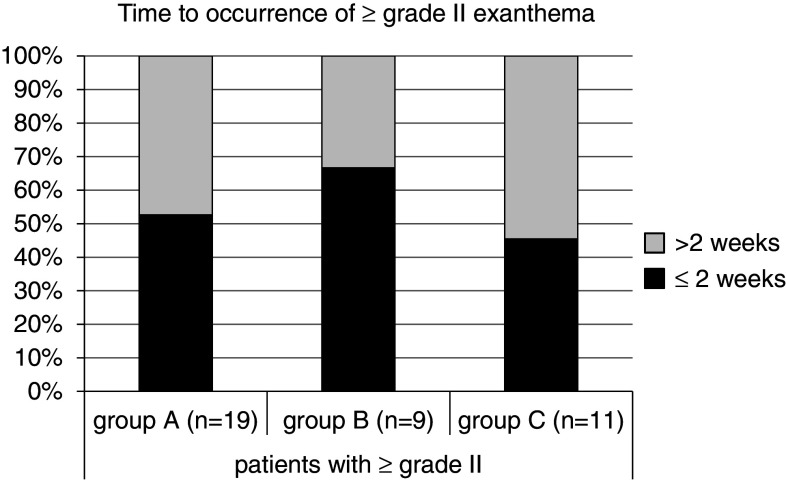
Time to occurrence of ≥grade II exanthema. No significant difference between the three cohorts exits in terms of time to first exanthema occurrence

During the first 12 weeks of therapy with cetuximab, 19/20 (95 %) patients in the historic cohort (group A) developed a skin exanthema: One patient (5 %) developed a grade IV° exanthema, 5 patients (25 %) experienced a grade III°, and 13 patients (65 %) a grade II° exanthema. Only one patient did not show clinical signs of exanthema (Fig. 3). Forty percent discontinued cetuximab therapy due to side effects (Fig. 4). Time to onset ranged from 1 to 4 weeks, and average time to onset was 14.7 days (Fig. 5).

In the second cohort receiving a reactive skin protocol (group B), all patients developed a skin exanthema (15/15; 100 %) within the first three months of cetuximab application: Two patients (13 %) developed a grade III° exanthema, eight patients (53 %) experienced a grade II° exanthema, and five patients (33 %) a grade I° exanthema (Fig. 3). Time to onset ranged from 1 to 4 weeks with an average time to onset of 13.2 days (Fig. 4). No patient had to discontinue cetuximab therapy (Fig. 5). No skin protocol-associated adverse events occurred. No patient terminated the in-house reactive skin protocol.

During the first 12 weeks of therapy with cetuximab in the third cohort receiving a prophylactic regimen (group C), all patients developed a skin exanthema (15/15; 100 %): One patient (7 %) developed a grade IV° exanthema, and one patient (7 %) developed a grade III° exanthema, while 9 patients (60 %) experienced a grade II° exanthema and four patients (27 %) a grade I° exanthema (Fig. 3). Time to onset ranged from 1 to 4 weeks, and average time to onset was 13.9 days (Fig. 5). One patient had to discontinue cetuximab therapy (Fig. 4).

A comparison of maximum exanthema (grade 0, I versus grade II, III, IV) in the three cohorts showed a significant difference between the historic cohort and the “reactive treatment” cohort (*p* = 0.027). Similar results exist between “historic” cohort group A and the “prophylactic treatment” cohort (group C; *p* = 0.069). However, there exists no significant difference between group B and C (*p* = 0.69).

## Discussion

To our knowledge, this is the first study comparing a reactive skin protocol with a prophylactic skin therapy in cetuximab-treated patients. Although two-third of the patients in the “reactive treatment” cohort developed a symptomatic grade ≥II° exanthema shortly after initiation of a cetuximab therapy, all patients were stabilized and had either no or only a very light grade I° exanthema after initiation of the reactive skin therapy. Note worthy is that the “prophylactic treatment” cohort showed equal effective but no superior results in preventing toxicity grade ≥II. Thus, a prophylactic use of topical skin and oral doxycycline treatment does not seem to improve efficacy but might be easier to handle in everyday practice and might also improve compliance. However, patients will have a higher intake of medication without superior results. As also presented earlier by our group, the use of the simple reactive skin protocol presented herein can prevent the exacerbation of a cetuximab-induced follicular acneiform exanthema. The application of this protocol prevented a cetuximab dose reduction or cessation in patients at risk. Protocol-induced adverse effects were not observed.

Only few reports about the therapeutic options of anti-EGFR skin exanthemas are published. In most cases, prophylactic approaches were chosen. The prophylactic application of oral antibiotics (tetracycline or minocycline) alone was effective for the early phase of the exanthema during the first month of anti-EGFR treatment (Scope et al. 2007; Jatoi et al. 2008).

A decrease in the incidence of grade ≥II° exanthemas was noted. In addition, tetracycline and minocycyline-treated patients reported other favorable symptomatic effects, including less itching, less burning and stinging, and less skin irritation compared to patients treated with placebo. However, these positive effects vanished after longer application. Similarly, topical usage of pimecrolimus, a calcineurin inhibitor, did not result in symptomatic relief or improvement of the severity of the cetuximab-induced exanthema (Scope et al. 2009). As anti-EGFR strategies, such as cetuximab, are part of a long-term cancer therapy, isolated usage of oral antibiotics seems to be an insufficient approach and has to be seen critically.

The STEPP study combined oral doxycycline with topical hydrocortisone (1 %) in a prophylactic setting (Lacouture et al. 2010). This approach resulted in a dramatic reduction in more severe exanthema and prolongation of time to onset of grade ≥II° exanthemas. However, this study has been often criticized, as all patients randomized in the prophylaxis arm had been exposed to topical hydrocortisone.

In our reactive skin protocol setting, only 13 % of patients, namely those who developed a grade III° exanthema, actually needed topical corticoid for a short period of time (maximum 3 weeks). Thus, it can be hypothesized that many patients participating in the STEPP study might have been exposed to hydrocortisone, unnecessarily.

The relevant limitation of our study is its retrospective setting and the number of patients treated. Thus, it is difficult to derive any validated clinical recommendations resulting from this report. A prospective study will be necessary in order to confirm our observations. Thus, there is a compelling need to continue and conduct research on how best to prevent and palliate exanthemas that occur from anti-EGFR therapy.
